# Addition of FFRct in the diagnostic pathway of patients with stable chest pain to reduce unnecessary invasive coronary angiography (FUSION): Rationale and design for the multicentre, randomised, controlled FUSION trial

**DOI:** 10.1007/s12471-022-01711-w

**Published:** 2022-08-17

**Authors:** S. P. Sharma, A. Hirsch, M. G. M. Hunink, M. J. M. Cramer, F. A. A. Mohamed Hoesein, C. A. Geluk, G. Kramer, J. W. C. Gratama, R. L. Braam, P. M. van der Zee, W. Yassi, S. L. Wolters, C. Gürlek, G. Pundziute, R. Vliegenthart, R. P. J. Budde

**Affiliations:** 1 Department of Cardiology, Erasmus Medical Centre, University Medical Centre Rotterdam, Rotterdam, The Netherlands; 2 Department of Radiology and Nuclear Medicine, Erasmus Medical Centre, University Medical Centre Rotterdam, Rotterdam, The Netherlands; 3 Department of Epidemiology and Biostatistics, Erasmus Medical Centre, University Medical Centre Rotterdam, Rotterdam, The Netherlands; 4 Centre for Health Decision Sciences, Harvard T.H. Chan School of Public Health, Boston, USA; 5 Department of Cardiology, University Medical Center Utrecht, Utrecht, The Netherlands; 6 Department of Radiology, University Medical Center Utrecht, Utrecht, The Netherlands; 7 Department of Cardiology, Martini Hospital Groningen, Groningen, The Netherlands; 8 Department of Radiology, Martini Hospital Groningen, Groningen, The Netherlands; 9 Department of Radiology, Gelre Hospital, Apeldoorn, The Netherlands; 10 Department of Cardiology, Gelre Hospital, Apeldoorn, The Netherlands; 11 Department of Cardiology, St Jansdal Hospital, Harderwijk, The Netherlands; 12 Department of Radiology, Adrz Hospital, Goes, The Netherlands; 13 Department of Cardiology, Adrz Hospital, Goes, The Netherlands; 14 Department of Cardiology, University Medical Centre Groningen, Groningen, The Netherlands; 15 Department of Radiology, University Medical Centre Groningen, Groningen, The Netherlands

**Keywords:** Angina, Stable, Computed tomography angiography, Coronary angiography, Coronary artery disease, Fractional flow reserve, Myocardial

## Abstract

**Background:**

Coronary computed tomography angiography (CCTA) is widely used in the diagnostic work-up of patients with stable chest pain. CCTA has an excellent negative predictive value, but a moderate positive predictive value for detecting coronary stenosis. Computed tomography-derived fractional flow reserve (FFRct) is a non-invasive, well-validated technique that provides functional assessment of coronary stenosis, improving the positive predictive value of CCTA. However, to determine the value of FFRct in routine clinical practice, a pragmatic randomised, controlled trial (RCT) is required. We will conduct an RCT to investigate the impact of adding FFRct analysis in the diagnostic pathway of patients with a coronary stenosis on CCTA on the rate of unnecessary invasive coronary angiography, cost-effectiveness, quality of life and clinical outcome.

**Methods:**

The FUSION trial is a prospective, multicentre RCT that will randomise 528 patients with stable chest pain and anatomical stenosis of ≥ 50% but < 90% in at least one coronary artery of ≥ 2 mm on CCTA, to FFRct-guided care or usual care in a 1:1 ratio. Follow-up will be 1 year. The primary endpoint is the rate of unnecessary invasive coronary angiography within 90 days.

**Conclusion:**

The FUSION trial will evaluate the use of FFRct in stable chest pain patients from the Dutch perspective. The trial is funded by the Dutch National Health Care Institute as part of the research programme ‘Potentially Promising Care’ and the results will be used to assess if FFRct reimbursement should be included in the standard health care package.

**Supplementary Information:**

The online version of this article (10.1007/s12471-022-01711-w) contains supplementary material, which is available to authorized users.

## Background

In the Netherlands, 180,000 new patients present with stable chest pain—the most common symptom of coronary artery disease (CAD)—annually [1]. In the past few years, the preferred diagnostic strategy in these patients has evolved from a focus on functional testing to coronary computed tomography angiography (CCTA) [2, 3]. Treating cardiologists in the Netherlands are recommended to apply a more consistent management by using a single non-invasive first-line test for diagnosing CAD due to current variation in clinical practice [1]. Implementation of CCTA in the work-up of patients with stable chest pain, however, may lead to unnecessary invasive testing, because CCTA has the ability to accurately rule out CAD [4, 5], but not the ability to correctly predict a haemodynamically significant stenosis on invasive coronary angiography (ICA) [6].

Computed tomography-derived fractional flow reserve (FFRct) may overcome the shortcomings of CCTA. FFRct is a non-invasive method that uses the already acquired CCTA images as the basis to calculate coronary FFR values as they would be expected if measured invasively (see Fig. S1 in Electronic Supplementary material). FFRct applies computational fluid dynamics and deep-learning image-based modelling to estimate rest and hyperaemic coronary flow and pressure from CCTA [7]. FFRct represents the ratio of maximal coronary blood flow through a stenotic artery to the blood flow in the hypothetical case that the artery is normal [8]. Early validation studies, assessing the diagnostic performance of FFRct with invasive FFR as the reference standard, are promising. On a per vessel analysis, the sensitivity is 80–88% and the specificity is 61–86% [9–11].

So far, observational data have shown that FFRct adds to the diagnostic strategy in patients with stable chest pain by reducing the rate of unnecessary ICA (defined as ICA showing unobstructed coronaries). The ADVANCE registry indicated that the availability of FFRct as an adjunct to CCTA would change the management of CAD in 66.9% of patients when compared with an initial CCTA-based treatment plan [12, 13]. This study also demonstrated the safety of patient management following the implementation of FFRct into the decision pathway, and the avoidance of invasive assessment in most patients with a negative FFRct [12, 13]. The PLATFORM study demonstrated a cancellation of ICA in 61% of CCTA + FFRct patients. No adverse clinical events occurred at 1 year in these patients, indicating safe deferral of ICA based on CCTA and FFRct [14]. The recent FORECAST trial showed that a strategy of CCTA with selective FFRct in stable angina patients did not differ significantly from standard clinical care pathways in cost and clinical outcomes, but did reduce the use of other non-invasive tests and ICA [15]. These studies suggest that FFRct can alter the management of patients and safely reduce the percentage of patients referred for ICA without significant CAD. An overview of the main findings of these current studies is presented in Tab. 1.

**Table 1:** Main characteristics of the studies on the use of FFRct in patients suspected with CAD

Study, year	First author	Study design	Sample size, *n*	Main results
*The ADVANCE registry, 2018, 2020*	Fairbairn et al.[13] and Patel et al. [12]	Pragmatic, prospective, international, multicentre registry of patients being investigated for clinically suspected CAD with documented stenosis of at least 30% on coronary CTA	5083	– FFRct modified treatment recommendation in 66.9% of the patients when compared with CCTA alone, was associated with less negative ICA, predicted revascularisation, and identified subjects at low risk of adverse events through 90 days. – 1-year outcomes show low rates of events in all patients, with less revascularisation and a trend toward lower MACE and significantly lower cardiovascular death or MI in patients with a negative FFRct compared with patients with abnormal FFRct values
*The PLATFORM study, 2015, 2016*	Douglas et al. [14, 23]	Prospective, consecutive cohort study with a comparative effectiveness observational design in symptomatic outpatients without known CAD, but with an intermediate likelihood of obstructive CAD, whose physician had planned non-emergent, non-invasive, or invasive cardiovascular testing to evaluate suspected CAD	584	– Among those with intended ICA (FFRct-guided = 193; usual care = 187), no obstructive CAD was found at ICA in 24 (12%) in the CTA/FFRct arm and 137 (73%) in the usual care arm. Invasive coronary angiography was cancelled in 61% after receiving CTA/FFRct results. Clinical event rates within 90 days were low in usual care and CTA/FFRct arms. – 1-year outcomes show that in patients with planned ICA, care guided by CTA and selective FFRct was associated with equivalent clinical outcomes and quality of life, and lower costs, compared with usual care over 1‑year follow-up
*The FORECAST trial, 2021*	Curzen et al. [15]	Open-label, multicentre, randomised, controlled clinical trial. Patients presenting at RACP, who require a cardiac test, were randomised to either routine care (management strategy based on NICE Chest Pain of Recent Onset Guidance—CG95) or CCTA with selective FFRct group in a 1:1 ratio	1400	A strategy of CCTA with selective FFRct in patients with stable angina did not differ significantly from standard clinical care pathways in cost or clinical outcomes, but did reduce the use of non-invasive testing and ICA, which was 22% lower in the experimental group
*Sub-study of the CRESCENT I and II trials, 2020*	Nous et al. [24]	Secondary analysis using the CCTA data. Observational cohort study, which included patients with suspected CAD and a ≥ 50% stenosis on CCTA who had been randomised to cardiac CT in the CRESCENT I trial and II trials	372	The availability of FFRct would have reduced the number of patients requiring additional testing by 57%-points compared with CCTA alone. The initial management strategy would have changed for 30 patients. Reserving ICA for patients with a FFRct ≤ 0.80 would have reduced the number of ICA following CCTA by 13%-points

*CAD* coronary artery disease, *CCTA* coronary computed tomography angiography, *FFRct* computed tomography-derived fractional flow reserve, *ICA* invasive coronary angiography, *RACP* rapid access chest pain clinic

Prior FFRct studies involved populations of various countries with different work-up and were based on a non-randomised design with the exception of the FORECAST trial [13, 14]. There are no prospective data on the real-world use of FFRct in the Dutch population of stable chest pain patients. On top of that, there are no trials that randomise patients who have undergone CCTA to standard care or FFRct-guided care based on the presence of > 50% stenosis on CCTA.

The ‘Addition of FFRct in the diagnostic pathway of patients with stable chest pain to reduce unnecessary invasive coronary angiography’ (FUSION) trial is a multicentre, randomised, controlled trial that investigates the impact of adding FFRct analysis in the diagnostic pathway of stable chest pain patients with a 50–90% coronary stenosis on CCTA, on the rate of unnecessary ICAs, the cost-effectiveness, quality of life and clinical outcomes.

The FUSION trial is sponsored by the Dutch Ministry of Health and the Dutch National Health Care Institute (*Zorginstituut Nederland*) as part of the research programme ‘Potentially Promising Care’ (‘Veelbelovende Zorg’) that aims to assess medical technology to determine if it can be reimbursed in the standard health care package.

## Methods

### *Study design and population*

**Fig. 1:**
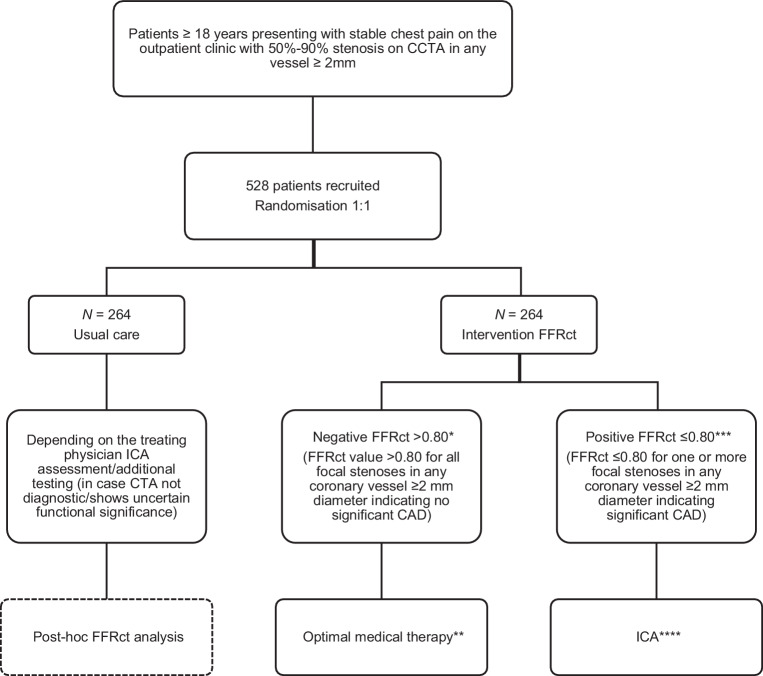
Flow chart of the study design. * Clinicians are strongly discouraged from referring these patients for ICA. ** Patients are expected to be treated medically, but there may be instances whereby the clinician overrules this decision. *** Clinicians are encouraged to schedule ICA if coronary revascularisation is considered advisable. **** Decision for ICA depends on symptomatology, stenosis and FFRct location, extent of CAD, number of coronary arteries involved, and comorbidities. In some cases, one may first treat the patient medically to see if the complaints of chest pain disappear. *CAD* coronary artery disease, *CCTA* coronary computed tomography angiography, *FFRct* computed tomography-derived fractional flow reserve, *ICA* invasive coronary angiography

The FUSION trial is an investigator-initiated, multicentre, randomised, controlled trial in the Netherlands enrolling 528 patients with stable chest pain and 50–90% stenosis in at least one coronary artery ≥ 2 mm on CCTA. CCTA is performed for clinical indications, therefore CCTA itself is not part of the study procedures. The inclusion and exclusion criteria are presented in Tab. 2. Patients will be randomised on a 1:1 basis to receive FFRct-guided care or usual care, and will be followed up for a minimum of 1 year. Fig. 1 provides a flow chart illustrating the overview of the study design. The trial will start in 7 Dutch hospitals: Erasmus Medical Centre, University Medical Centre Groningen, University Medical Centre Utrecht, Adrz Hospital, Gelre Hospital, St. Jansdal Hospital, and Martini Hospital Groningen. Additional centres may join after the trial commences.

**Table 2:** Inclusion and exclusion criteria

Inclusion criteria	Exclusion criteria
Age ≥ 18 years	Inability to provide informed consent
Stable chest pain	Unstable angina/acute coronary syndrome
Underwent CCTA with ≥ 50% but less than 90% stenosis in at least one major epicardial vessel with a diameter ≥ 2 mm	Unstable clinical status
	Expected inability to complete follow-up and comply with follow-up aspects of the protocol
	History of coronary revascularisation
	Non-invasive or invasive diagnostic testing for CAD within the past 12 months (with the exception of exercise ECG)
	Unsuitable for revascularisation if required (for example due to comorbidities or anatomical features)
	Poor CT quality with expected inability to perform FFRct analysis

*CAD* coronary artery disease, *CCTA* coronary computed tomography angiography, *ECG* electrocardiogram, *FFRct* computed tomography-derived fractional flow reserve

### Study procedures

#### The intervention group (FFRct group)

In the intervention group, FFRct analysis will be performed based on the available CCTA data, without the need of additional imaging, radiation or medication. FFRct will be performed by HeartFlow, Inc. (Redwood City, California, United States) in all patients. A secure web transfer portal will be established with each site allowing transfer of the CCTA data to HeartFlow for FFRct analysis. The FFRct output will be returned to the investigating site within 1 working day [9]. Subsequently, the patient management strategy is determined with incorporation of the FFRct results (Fig. 1). In principle, patients with a negative FFRct (FFRct of > 0.80) should undergo conservative management. Patients with a positive FFRct (FFRct ≤ 0.80 for one or more focal stenoses in any coronary artery ≥ 2 mm) should undergo subsequent ICA.

#### The control group (usual care group)

All patients in the control group will undergo usual care. In case of an anatomical stenosis on CCTA, this often leads to ICA and invasive FFR measurement. The indication for ICA and revascularisation is at the discretion of the treating physician. It is recommended that the treating physician follows the European Society of Cardiology guidelines for the diagnosis and management of chronic coronary syndrome [2].

#### Post-hoc FFRct analysis in usual care group

Since in the intervention group, patients with FFRct > 0.80 will in principle not undergo ICA, false negative FFRct cases remain unidentified. Therefore, FFRct will also be performed in the usual care group (post-hoc FFRct), but will neither be revealed to the clinical site nor influence the diagnostic pathway. The treating clinician will be blinded to these FFRct results. This way, we can indirectly gauge the number of false negative FFRct findings retrospectively in patients who underwent ICA in the control group without influencing patient management in this group. A false negative is defined as negative FFRct result with an ICA showing haemodynamically significant stenosis on invasive FFR.

Data to be collected at baseline, 90 days and 1 year are presented in Electronic Supplementary Material.

### Study endpoints

The primary endpoint is the rate of unnecessary ICA occurring within 90 days in both groups as determined on a per patient level. Unnecessary ICA is defined as any ICA without haemodynamically significant CAD. The leading indicator for the evaluation of significant CAD is the functional measurement (FFR/iFR). If functional measurements are not available, then significant CAD is indicated by quantitative coronary angiography. Primary and secondary endpoints definitions are listed in Fig. S2 in Electronic Supplementary Material.

### Statistical analysis

#### Sample size calculation

We estimate that 28–41% of the patients who undergo ICA after CCTA do not have a haemodynamically significant stenosis [16, 17]. With the addition of FFRct we expect that the number of unnecessary ICA procedures will decrease by 33%. To reach an 80% power, a 0.05 significance level, and assuming a 10% drop-out rate, 528 patients need to be included in the trial (264 in each group).

#### Data analysis

Statistical analysis will be performed using SPSS computer software and R software where necessary. Descriptive statistics will be used for presenting data. The analysis of the primary outcome and the secondary outcomes will use an intention-to-treat approach. A *p*-value of < 0.05 will be considered statistically significant.

In a logistic regression model we will analyse the association between the primary outcome and the random group assignment (FFRct versus usual care). The analysis will be performed both unadjusted and adjusted. Since we know from previous studies that age, sex, type of chest pain, and coronary artery calcium score are associated with haemodynamically significant CAD, we will adjust for these factors [18]. In a third model we will also analyse the effect of each participating centre with adjustment for the mentioned factors.

For the secondary endpoint, unnecessary ICA at 1 year and the other secondary endpoints similar statistical analyses will be performed as for the primary endpoint.

#### Cost-effectiveness analysis

We will perform a cost-utility analysis in accordance with the Dutch guidelines for economic evaluations in health care (*Zorginstituut 2016*) from the societal and the health care perspective. A cost (EUR) and outcomes (quality-adjusted life-year) comparison between the FFRct and the usual care groups as observed in the RCT for the 1‑year time frame will be made. Next, a state-transition model will be built to calculate the cost-effectiveness of FFRct considering a long-term time horizon. In this model we will synthesise data from the RCT with the post-hoc results of FFRct in the usual care group and literature data.

### Trial structure, registration and organisation

The study complies with good clinical practice in accordance with the Declaration of Helsinki and the laws and regulations applicable in the Netherlands, including the General Data Protection Regulation, as the clinical trial has been approved by the appropriate Medical Research Ethics Committee and Review Board (Erasmus MC, MEC-2021-0189). The clinical trial was registered under number NL76830.078.21 (NCT05174247, clinical trial registration number) on 29 December 2021. The trial started enrolment on 28 July 2021.

## Discussion

This multicentre, randomised clinical trial will evaluate the impact of adding the FFRct analysis to CCTA in the diagnostic pathway of stable chest pain patients with CCTA-derived stenosis on the rate of unnecessary ICA, cost-effectiveness, quality of life, and clinical outcomes from a Dutch perspective.

FFRct is a non-invasive technique that may change the way stable angina is diagnosed and managed. Patients who undergo ICA after CCTA often do not have a haemodynamically significant stenosis, which leads to 28–41% unnecessary ICAs in the Dutch population [16, 17]. By deriving coronary physiology from CCTA data, FFRct can be used to increase the diagnostic yield of the CCTA and, consequently, reduce the number of unnecessary ICAs as well as costs [13, 14].

In addition, FFRct may add to a proper selection of patients who may benefit from a revascularisation procedure [13]. FFRct has been validated against invasive FFR, which is an essential invasive procedure guiding revascularisation in stable CAD by investigating the haemodynamic significance of epicardial coronary stenoses (FAME trial) [9–11, 19]. Although ICA with FFR could be performed in the management of patients with stable chest pain, the invasive approach has its challenges; not only the complications inherent to invasive testing, but also the inevitable patient discomfort and costs. Determining FFR using CCTA could provide both anatomical and physiological assessment of the coronary circulation without cardiac catheterisation.

To evaluate the impact of FFRct in the FUSION trial, selection of an appropriate target population is necessary. Given the high negative predictive value of CCTA, only little added benefit of FFRct is expected in patients without suspected obstructive disease on CCTA. On the other hand, functional evaluation is also not required in case of a very high grade stenosis (> 90%) on CCTA, because the probability of this stenosis to be haemodynamically significant on ICA is very high [8, 20]. Therefore, we will only include patients with a 50–90% stenosis on CCTA.

Similar to the design of the CRESCENT trial [17], the FUSION trial design is based on improving the diagnostic strategy of stable angina pectoris patients and not merely on identifying which diagnostic test is superior. In the FUSION trial, we focus on patients in whom we expect FFRct to be most appropriate, namely patients with intermediate anatomic stenosis. On top of that, we have designed our study in such a way that we can estimate the number of false negative FFRct results and determine the effects of missing CAD diagnosis, by performing a post-hoc FFRct analysis in the control group based on the existing CCTA in each patient. The patients in the control group undergo routine care, which is not based on FFRct but, mostly, on ICA. This ensures that we can determine how often the FFRct results would have led to a false negative result. In the intervention group, patients with FFRct > 0.80 will include both the true and false negatives, and we will assess the outcome of these patients. These results combined will provide solid data on how often false negatives occur and what the consequences are. We will be able to make an assumption on the maximum risk these patients have for developing major adverse cardiac events in 1 year.

Major adverse cardiac events is not selected as the primary endpoint, as it would require a very high number of patients to be included in the trial. Since we can make accurate assumptions of the risk of major adverse cardiac events in the study population—and unnecessary ICAs is a clinically relevant endpoint—we have chosen the latter as the primary endpoint.

The FUSION trial has been designed to investigate the role of FFRct in the diagnostic strategy of patients with stable chest pain after CCTA and does not have the potential to answer questions about optimal treatment strategy in patients with symptomatic CAD. This was subject of the ISCHEMIA trial, which results support that the vast majority of patients with CAD (in a selected population of patients with an abnormal functional test) can be treated with an initial conservative strategy of medical therapy alone [21]. These results do not undermine the prominent role of functional testing in the management of stable chest pain patients, whether this should be based on functional testing or based on FFR testing, such as in the FUSION trial.

First of all, it remains useful to confirm a clinical diagnosis in patients with quality-of-life-limiting symptoms. Secondly, as the ORBITA trial demonstrated, having symptoms and an anatomic stenosis is insufficient to detect the patients for whom revascularisation will improve quality of life [22]. On top of that, ISCHEMIA targeted a specific patient population, as most patients referred for further testing in the current era would have been too low risk to be included in the trial, but also high-risk patients (unacceptable level of angina despite maximal medical therapy, left main disease on CCTA, ejection fraction < 35%) were excluded [21].

In conclusion, the FUSION trial is designed to evaluate whether the use of FFRct could decrease unnecessary ICA in patients with stable chest pain in both academic and peripheral hospitals with multiple CCTA vendors. The FUSION trial aims to provide a new strategy in Dutch health care for the diagnostic workup of stable chest pain. As the FUSION trial is part of the research programme ‘Potentially Promising Care’ of the Dutch National Health Care Institute, the results of the study will be used for a fast-track procedure to assess if FFRct reimbursement can be included in the standard health care package.

## Supplementary Information

**Fig. S1 **CCTA, FFRct and invasive coronary angiography assessment. A 66-year-old male patient, presenting at the outpatient clinic with atypical chest pain. (A) Coronary computed tomography (CCTA) showed 70% stenosis in the proximal right coronary artery (RCA). (B) CCTA-derived fractional flow reserve (FFRct) suggested no haemodynamically significant stenosis in the RCA (FFRct value 0.88). (C) This was confirmed by invasive coronary angiography (ICA) with invasive FFR measurement of the RCA (FFR value 0.89).

Overview of data collected at baseline, 90-day follow-up and 1‑year follow-up

**Fig. S2 **Primary and secondary endpoints. *CAD* coronary artery disease, *FFR* fractional flow reserve, *FFRct* computed tomography-derived fractional flow reserve, *ICA* invasive coronary angiography, *iFR* instant flow reserve, *QCA* quantitative coronary analysis
