# Supplementary figures and images for: Addition of FFRct in the diagnostic pathway of patients with stable chest pain to reduce unnecessary invasive coronary angiography (FUSION): Rationale and design for the multicentre, randomised, controlled FUSION trial
Source: Neth Heart J. 2022 Aug 17;31(2):52–60. doi: 10.1007/s12471-022-01711-w (PMC9892409; doi:10.1007/s12471-022-01711-w)

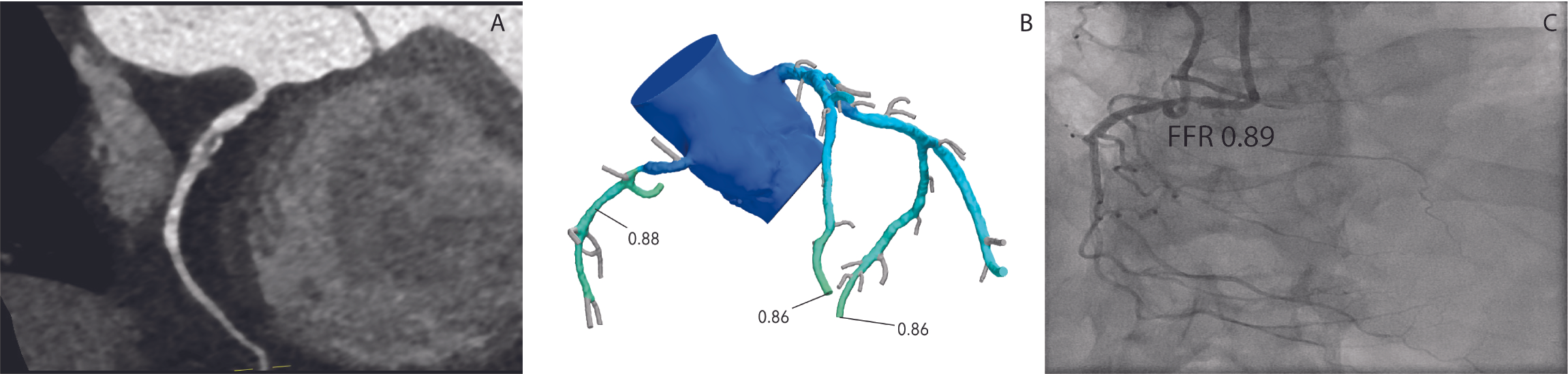

Supplement: Supplementary file 1 — Fig. S1 CCTA, FFRct and invasive coronary angiography assessment. A 66-year-old male patient, presenting at the outpatient clinic with atypical chest pain. (A) Coronary computed tomography (CCTA) showed 70% stenosis in the proximal right coronary artery (RCA). (B) CCTA-derived fractional flow reserve (FFRct) suggested no haemodynamically significant stenosis in the RCA (FFRct value 0.88). (C) This was confirmed by invasive coronary angiography (ICA) with invasive FFR measurement of the RCA (FFR value 0.89). [file 12471_2022_1711_MOESM1_ESM.tiff]
