# Supplementary material for: Addition of FFRct in the diagnostic pathway of patients with stable chest pain to reduce unnecessary invasive coronary angiography (FUSION): Rationale and design for the multicentre, randomised, controlled FUSION trial
Source: Neth Heart J. 2022 Aug 17;31(2):52–60. doi: 10.1007/s12471-022-01711-w (PMC9892409; doi:10.1007/s12471-022-01711-w)
Supplement: Supplementary file 2 — Overview of data collected at baseline, 90-day follow-up and 1‑year follow-up [file 12471_2022_1711_MOESM2_ESM.docx]

**Overview of data collected at baseline, 90-day follow-up and 1-year follow-up**

|  | **Baseline** | **90-day follow-up** | **1-year follow-up** |
| --- | --- | --- | --- |
| **Coronary computed tomography angiography (CCTA)** | **X** |  |  |
| **Demographics** | **X** |  |  |
| **Baseline patient characteristics** | **X** |  |  |
| **Medical history** | **X** |  |  |
| **Physical exam** | **X** |  |  |
| **Electrocardiogram (ECG)** | **X** |  |  |
| **Laboratory tests*** | **X** |  |  |
| **Fractional flow reserve derived from CT (FFRct) analysis** | **X**  **(test group and control group**)** |  |  |
| **3 Quality of Life questionnaires** | **X** | **X^1^** | **X^1^** |
| **Medical questionnaire^a^** |  | **X^1^** | **X^1^** |
| **Clinical follow-up** |  | **X^2^** | **X^2^** |

***1 = questionnaires sent by post or digital mail. Seattle Angina Questionnaire (SAQ), 36-Item Short Form Survey (SF36), EuroQOL 5d-5L(EQ-5D-5L)***

***2 = contact by phone or visit at outpatient clinic***

***a = questionnaire to enquire about clinical events for follow-up purposes***

**** If available, three weeks before or after randomisation may be included***

***** FFRct results will be blinded to the treating physician***
