# Supplementary material for: Addition of FFRct in the diagnostic pathway of patients with stable chest pain to reduce unnecessary invasive coronary angiography (FUSION): Rationale and design for the multicentre, randomised, controlled FUSION trial
Source: Neth Heart J. 2022 Aug 17;31(2):52–60. doi: 10.1007/s12471-022-01711-w (PMC9892409; doi:10.1007/s12471-022-01711-w)
Supplement: Supplementary file 3 — Fig. S2 Primary and secondary endpoints. CAD coronary artery disease, FFR fractional flow reserve, FFRct computed tomography-derived fractional flow reserve, ICA invasive coronary angiography, iFR instant flow reserve, QCA quantitative coronary analysis [file 12471_2022_1711_MOESM3_ESM.docx]

No anatomical narrowing of ≥70% in any coronary vessel ≥2 mm diameter, for the left main artery there should be no anatomical narrowing of ≥50%

Secondary endpoints:
Unnecessary ICA within 1 year

Major Adverse Cardiac Events
(composite endpoint of all-cause mortality,
non-fatal myocardial infarction and unplanned hospitalisation leading to urgent revascularisation) within 90 days and 1 year

Number of additional non-invasive tests for CAD assessment within 90 days and 1 year

All coronary revascularisations (planned/unplanned) within 90 days and 1 year

Quality of life within 90 days and 1 year

Cardiovascular death within 90 days and 1 year

Complications during and after ICA within 90 days and 1 year

Non-fatal stroke within 90 days and 1 year

Cost-effectiveness and budget impact analysis within 1 year

Visual assessment

No

Yes

No

Yes

QCA available?

FFR/iFR available?

No anatomical narrowing of ≥50% on QCA in any coronary vessel ≥2 mm diameter

No FFR measurement of ≤0.80 or no iFR ≤0.89 in any coronary vessel ≥2 mm diameter

ICA without haemodynamically significant CAD is functionally defined as:

Primary endpoint:
Unnecessary ICA within 90 days, defined as any ICA without haemodynamically significant CAD (leading indicator to evaluate significant CAD is FFR/iFR, if it is not available then CAD is indicated by QCA or ultimately by visual assessment)
